# Mehr Kooperation von Start-ups und Mittelstand als Chance für Digitalisierung und Innovationen

**DOI:** 10.1007/s10273-021-2922-5

**Published:** 2021-05-17

**Authors:** Klaus-Heiner Röhl, Barbara Engels

**Affiliations:** 1 Kompetenzfeld Digitalisierung, Strukturwandel und Wettbewerb, Institut der deutschen Wirtschaft Köln e.V., Georgenstraße 22, 10117 Berlin, Deutschland; 2 Industrieökonomik und Wettbewerb, Institut der deutschen Wirtschaft Köln e.V., Konrad-Adenauer-Ufer 21, 50668 Köln, Deutschland

**Keywords:** L14, L23, L26

## Abstract

Die Corona-Krise hat die Defizite Deutschlands in der Digitalisierung von Wirtschaft und Gesellschaft schlagartig offengelegt. Die Zusammenarbeit von etabliertem Mittelstand und innovativen Start-ups — oft mit digitalen Geschäftsmodellen — bietet dabei große Chancen für die Beteiligten und die Wirtschaft insgesamt. Auch könnte das demografiebedingt nachlassende Wachstumspotenzial der deutschen Wirtschaft steigen.

## Abstract (en)

The Coronavirus crisis clearly exposed Germany’s deficits in the digitalisation of the economy and society. The cooperation between established SMEs and innovative start-ups offers considerable opportunities for the respective companies and the German economy. New business models can be adopted and innovative products can be jointly developed. In particular, the digitalisation of the ‘German Mittelstand’ could receive a boost through collaboration with digital start-ups. This could lead to an increase in the demographically induced declining growth potential of the German economy.

Deutschland ist bislang kein Pionier bei der Entwicklung der Digitalwirtschaft und dem Einsatz künstlicher Intelligenz (KI); besonders der Mittelstand setzt digitale Technologien eher verhalten ein (Büchel et al., 2020). Die Digitalisierung des Mittelstands könnte durch Kooperationen mit digitalen Start-ups einen Schub erhalten (Definitionen vgl. Kasten 1); neue Geschäftsmodelle und Produkte können gemeinsam entwickelt werden. Anhand verfügbarer Studien wurde der Stand der Kooperationsbeziehungen untersucht,[1] und basierend auf Expert:inneninterviews werden Potenziale zur Stärkung der Beziehungen zwischen etabliertem Mittelstand und der wachsenden Start-up-Szene aufgezeigt.

In den letzten Jahren haben Start-ups nicht nur digitale Geschäftsmodelle, etwa in der Plattformökonomie (Demary und Rusche, 2018), entwickelt, sondern auch zunehmend an digitalen Lösungen für bestehende Modelle gearbeitet. Dabei besitzt die Digitalisierung zahlreiche Facetten, die in komplexer Art miteinander verwoben sind:

- die weitere Automatisierung der Produktion,
- die Digitalisierung von Schnittstellen zu anderen Unternehmen in der Wertschöpfungskette,
- die Nutzung digitaler Technologien, um etablierte Geschäftsmodelle weiterzuentwickeln, und
- die Entwicklung vollkommen neuer, disruptiver digitaler Geschäftsmodelle.

Trotz der wachsenden deutschen Start-up-Szene und ihrer Ausdehnung über die beiden langjährigen Cluster in Berlin und München hinaus werden die Möglichkeiten, die sich aus der Kooperation etablierter Firmen besonders im Mittelstand mit Start-ups ergeben, bislang nicht voll ausgenutzt.

## Vorteile und Potenziale der Kooperation

Viele der Potenziale, die neue Technologien und Geschäftsmodelle sowie Digitalisierung bieten, ergeben sich aus der Kooperation mit anderen Unternehmen, oft branchenübergreifend. Digitale Start-ups bieten technologisches Know-how oder Geschäftsmodelle, die für kleine und mittlere Unternehmen (KMU) besonders interessant sein können. Den Kapazitäten von KMU, Digitalkompetenzen zu entwickeln, sind engere Grenzen gesetzt als es bei Großunternehmen der Fall ist. Durch die Digitalisierung der Wertschöpfungsketten im Rahmen der Industrie 4.0 verschwimmen die Grenzen zwischen traditioneller Industrie und Dienstleistungen. Viele Start-ups agieren in diesem Grenzbereich. Durch Kooperationen können etablierte Firmen von der Disruption, die von Start-ups ausgeht, profitieren.

Kooperationen und die Einbindung in Unternehmensnetzwerke bieten Vorteile für Produktion, Beschaffung und Vertrieb sowie für Innovationsprojekte. Innovative Unternehmen pflegen enge Beziehungen zu ihren Lieferanten und Kunden, aber auch zu Wettbewerbern, um die Entwicklung neuer Produkte und Prozesse zu beschleunigen

**Dr. Klaus-Heiner Röhl** ist Senior Economist beim Institut der deutschen Wirtschaft am Standort Berlin.

**Barbara Engels** ist Economist beim Institut der deutschen Wirtschaft am Standort Köln.

1 Basierend auf Engels und Röhl (2019) sowie Röhl und Engels (2020).

### Kasten 1
### **Definitionen**

In Anlehnung an die international übliche Definition bezeichnet „Start-up“ junge Unternehmen, die innerhalb der vergangenen zehn Jahre gegründet wurden, eine innovative Technologie oder ein neues Geschäftsmodell nutzen und ein hohes Wachstum anstreben.

Der Begriff Mittelstand wird hier weit gefasst. Eingeschlossen sind sowohl kleine und mittlere Unternehmen (KMU) im klassischen Sinne mit weniger als 250 Mitarbeiter:innen und einem maximalen Umsatz von 50 Mio. Euro als auch mittelständisch strukturierte Familienunternehmen (Institut für Mittelstandsforschung Bonn, o. D.), die diese Größengrenzen überschreiten.

Unter Digitalisierung wird der Wandel von einer analogen in eine digitale Form verstanden. Er reicht von der Idee, dem Auftrag, der Entwicklung und Produktion über die Auslieferung und das Recycling eines Produkts bis hin zu damit verbundenen Dienstleistungen. Entscheidend ist die Verfügbarkeit aller relevanten Informationen in Echtzeit durch Vernetzung aller an der Wertschöpfung beteiligten Akteur:innen und die Fähigkeit, aus den Daten den optimalen Wertschöpfungsfluss zu generieren (Lichtblau et al., 2015). Digitalisierung umfasst zudem die Entwicklung und Nutzung digitaler Geschäftsmodelle.

### Abbildung 1
### **Potenzielle Probleme in der Zusammenarbeit mit Start-ups aus Sicht der Familienunternehmen**

N = 213, Mehrfachantworten möglich

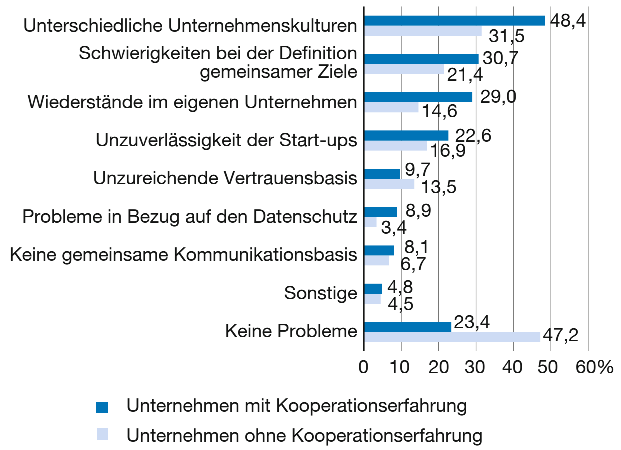

Quellen: Bundesverband der Deutschen Industrie (BDI) und Institut für Mittelstandsforschung Bonn (IfM) (2018, 7).

(Fritsch et al, 2020). Durch die Nutzung des im Netzwerk vorhandenen Know-hows gelingt es KMU, ihre Kompetenzen zu erweitern. Die Kenntnis des Partners und das Vertrauen in seine Fähigkeiten sind zentral für stabile Netzwerke. Dies ist einer der Gründe, warum trotz der Verbreitung digitaler Plattformen klassische Netzwerke und persönliche Kontakte bei der Anbahnung von Kooperationen zwischen Start-ups und KMU wichtig bleiben. Oft findet die Kooperation in regionalen Clustern oder innovativen Milieus statt (Lichtblau et al., 2005; Röhl, 2019).

Etablierte Mittelständler und Start-ups haben unterschiedliche Kompetenzprofile, die sich gegenseitig ergänzen können. Start-ups können neue Ideen leichter am Markt testen, während etablierte Firmen mit langjährigem Kundenstamm vorsichtiger agieren müssen. Bei der Implementierung neuer Technologien können KMU das digitale Know-how von Start-ups einbinden. Zudem kann der Mangel an qualifiziertem Personal (Anger et al., 2018) durch Kooperationen zum Teil kompensiert werden. Eng verbunden mit der Nutzung komplementärer Fähigkeiten ist der Zugang zu neuen Technologien und (Online-)Märkten bzw. Plattformen, den KMU durch die Kooperation mit Start-ups gewinnen können (Leitner et al., 2019). Im Gegenzug erhalten die Start-ups Zugang zu Netzwerken ihrer Partner und oft auch zu Kapital, das sie zur weiteren Expansion benötigen.

Innovationen sind eine Voraussetzung für den Erhalt der Wettbewerbsfähigkeit. Dies gilt besonders für industrielle Mittelständler, die im internationalen Wettbewerb stehen. Studien zeigen aber, dass sich die Innovationsausgaben der Wirtschaft zunehmend auf Großunternehmen ab 1.000 Beschäftigten konzentrieren, während der Anteil kontinuierlich innovierender KMU sinkt (Behrens et al., 2017).

## Mittelstand und Start-ups: unterschiedliche Welten?

Zu den Hürden gehören kulturelle Unterschiede, die sich aus der unterschiedlichen Geschäfts-, Markt- und Personalsituation sowie Entwicklungsstadien ergeben (Leitner et al., 2019). Der „Cultural Fit“ zwischen etablierten KMU und Start-ups scheint oft schlecht zu sein (Deloitte, 2017). In der Befragung des BDI-Familienunternehmenspanels (Bundesverband der Deutschen Industrie [BDI] und Institut für Mittelstandsforschung Bonn [IfM], 2018) gab fast ein Drittel der Unternehmen mit Kooperationserfahrung an, dass kulturelle Differenzen ein Problem darstellen – die mit Abstand häufigste Angabe (vgl. Abbildung 1).

Der deutsche Mittelstand besteht zu einem großen Teil aus Familienunternehmen mit langer Tradition, vor allem in der Industrie. Zwischen ihrer Unternehmenskultur und der von Start-ups scheint es große Unterschiede zu geben:

- Familienunternehmen zeichnen sich durch ein hohes Maß an Kontinuität in ihren Strukturen und Arbeitsweisen aus. Unternehmerische Umsicht ist essenziell, um über Jahrzehnte erfolgreich zu sein (Röhl, 2008). Start-ups hingegen sind auf rasante Entscheidungen und Disruption ausgerichtet (Leitner et al., 2019).

- Die Entwicklung vom regional agierenden KMU zum global aufgestellten Hidden Champion wird in Familienunternehmen vor allem durch einbehaltene Gewinne gemeistert, was sich in hohen und bis zur Corona-Krise steigenden Eigenkapitalquoten widerspiegelt (Deutsche Bundesbank, 2020). Start-ups hingegen sind auf externe Finanzierungsquellen angewiesen.

- Bei etablierten KMU handelt es sich meist um Unternehmen, die stark in ihrem regionalen Umfeld verankert sind und deren Inhaber-Geschäftsführer:innen eine ähnliche Mentalität haben wie die Menschen in ihrer oft ländlichen Heimatregion (Kempermann et al., 2020). Im Gegensatz dazu sind Start-up-Gründer:innen Digital Natives in einem urbanen Umfeld (Kollmann et al., 2018).

Mit neuen digitalen Geschäftsmodellen wie KI oder Plattformen stoßen Start-ups Disruptionen in der Wirtschaft an, wobei ein Scheitern oft als mögliche frühe Exit-Option in Betracht gezogen wird: War die Geschäftsidee nicht erfolgreich, wird das Start-up liquidiert, um mit den gewonnenen Erfahrungen Alternativen zu verfolgen (Röhl, 2019, 134; Organisation for Economic Cooperation and Development [OECD], 2013). Die Unsicherheit über das langfristige Bestehen des potenziellen Partners kann für etablierte Mittelständler ein Kooperationshindernis sein. Differenzen in der Unternehmenskultur könnten auch aus der Altersstruktur resultieren. 2013 war mehr als die Hälfte der mittelständischen Unternehmer:innen über 50 Jahre alt und ein gutes Fünftel bereits über 60 Jahre alt (KfW Research, 2015). Im Gegensatz dazu sind Start-up-Gründer:innen laut Start-up Monitor im Durchschnitt 36 Jahre alt (Kollmann et al., 2020). Der Altersunterschied von ca. einer Generation kann dazu beitragen, dass sich die Ansichten und Verhaltensmuster unterscheiden.

### Differenzen im Innovationsverhalten

Ein wichtiger Unterschied im Innovationsverhalten zwischen der Mehrheit der etablierten KMU und Start-ups lässt sich unter den Begriffen „inkrementelle Innovation“ versus „Disruption durch neue Technologien und Geschäftsmodelle“ fassen. Während grundlegende Innovationen eher zögerlich angegangen werden, konzentrieren sich gerade industrielle Mittelständler auf kontinuierliche, an den Kundenwünschen orientierte Verbesserungen von Produkten und Prozessen (IW Consult und Santiago, 2015; KfW Research, 2019).[2]

Das kundenorientierte Innovationsmodell hat zur Exportstärke der deutschen Industrie beigetragen und ist mitverantwortlich dafür, dass es in Deutschland über 1.300 Hidden Champions gibt (Röhl, 2018). Die langsame Adoption von Industrie-4.0-Technologien und die Schwierigkeiten, geeignete Fachkräfte zu finden, weisen jedoch auf die Grenzen der inkrementellen Innovationsstrategie hin (IW Consult, 2018). Um zukünftig im internationalen Wettbewerb bestehen zu können, muss die Fähigkeit zu Sprunginnovationen erhöht werden. Die Folgen der Corona-Krise erhöhen den Druck hierzu weiter. Daten zum Innovationsverhalten deutscher Unternehmen deuten aber in die Gegenrichtung; weniger KMU betreiben eigene Forschung und Entwicklung. Die rückläufigen Innovatorenquoten in Unternehmen mit bis zu 1.000 Beschäftigten laut ZEW-Innovationserhebung können als Alarmsignal für die Zukunftsfähigkeit der deutschen Industrie und des Wirtschaftsstandorts insgesamt gesehen werden (Röhl, 2018). Nicht zuletzt deshalb ist eine engere Zusammenarbeit zwischen Mittelständlern und innovativen Start-ups ein Weg, um digitale Innovationen und die Erschließung neuer Geschäftsfelder zu fördern.

### Räumliche Differenzen

Eine weitere potenzielle Hürde für die Zusammenarbeit zwischen Mittelstand und Start-ups ergibt sich aus ihren abweichenden geografischen Schwerpunkten, besonders im Industriesektor. Ein großer Teil der Start-ups ist in Ballungen angesiedelt, wobei Berlin und München als Standorte mit der größten Zahl innovativer Gründungen und den höchsten VC-Investitionssummen hervorstechen (EY, 2021). Der Mittelstand ist gleichmäßiger über das Land verteilt. Industrielle Mittelständler weisen Cluster in süd- und westdeutschen Regionen auf, die zum Teil ländlich sind. Abbildung 2 zeigt die führenden deutschen Gründerhochburgen und die regionalen Schwerpunkte des industriellen Mittelstands. Generell ist der Mittelstand trotz der Cluster räumlich weniger konzentriert als die Gründerszene.

München spielt als führender Standort für B2B-Start-ups der Hochtechnologie eine zentrale Rolle für die Industrie in Bayern. Positiv hat sich zuletzt auch die Start-up-Landschaft in Baden-Württemberg, das ebenfalls viele industrielle KMU aufweist, entwickelt. Inzwischen gehört die Region Stuttgart-Karlsruhe laut Deutschem Startup Monitor mit gut 6 % der 2018 verzeichneten Gründungen zu den Top 5 Standorten in Deutschland (Kollmann

2 Die Rolle von Kundenkooperationen bestätigen auch Auswertungen der Befragungswellen 2011 bis 2018 des IW-Zukunftspanels.

Abbildung 2

**Räumlicher Schwerpunkt von Start-ups und industriellem Mittelstand**

Anteile der fünf führenden Gründerzentren an erfassten Gründungen 2020 in % und Cluster industrieller KMU

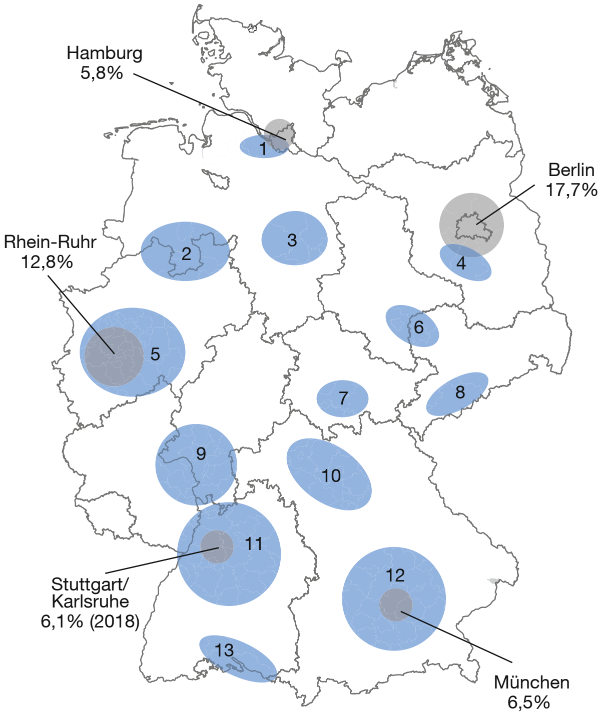

Führende Start-up-Regionen Cluster des industriellen Mittelstands

1 Hamburg und Umland
2 Münster-Osnabrück-Bielefeld
3 Hannover-Braunschweig-Wolfsburg
4 Berlin/südliches Umland
5 Rheinland-Westfalen
6 Leipzig-Halle
7 Zentral- und Südthüringen
8 Südwestsachsen
9 Südhessen-Rhein-Neckar
10 Franken
11 Baden-Württemberg (Nord)
12 Bayern
13 Bodensee

Quelle: T. Kollmann et al. (2020), eigene Erstellung.

et al., 2018, 23). Berlin als führendes Start-up-Zentrum weist einen unterdurchschnittlichen Bestand an mittelständischen Industrieunternehmen auf. Damit fehlt es an potenziellen Industriepartnern für die vielen Start-ups der Stadt: Allein 2019 wurden 564 neu gegründete Start-ups identifiziert (Institut für Innovation und Technik [IIT], 2020, 9). Viele Berliner Start-ups sind auf den KI-Bereich spezialisiert (Goecke und Thiele, 2018). Die Hauptstadtregion bietet somit ein hohes Potential für Kooperationen in diesem zukunftsweisenden Entwicklungsfeld.

In Interviews mit Expert:innen aus KMU, Start-ups und Verbänden wurde die hinderliche Wirkung der räumlichen Verteilung unterschiedlich bewertet. Die Rolle persönlicher Kontakte und damit räumlicher Nähe wurde aber überwiegend bestätigt. Die anfängliche Konzentration der Gründerszene in Berlin wird als eine Voraussetzung für eine erfolgreiche Clusterbildung gesehen. In jüngster Zeit entstehen immer mehr Gründungen im B2B-Bereich an anderen Standorten wie München, Stuttgart, Hamburg oder in den Ballungsräumen Nordrhein-Westfalens, sodass die räumliche Passung zwischen Start-ups und KMU zunimmt.

## Kooperationsentwicklung zwischen KMU und Start-ups: empirische Ergebnisse

Ein steigendes Interesse an Kooperationen zwischen Start-ups und KMU lässt sich an der wachsenden Zahl von Studien ablesen, die sich mit dem Thema beschäftigen. Etwa ein Drittel der 250 in einer RKW-Studie befragten KMU (Wallisch, 2018) hat bereits Kooperationserfahrungen gesammelt. Während digitale Plattformen bei der gezielten Partnersuche helfen können, erhöht räumliche Nähe die Wahrscheinlichkeit von Zufallskontakten. Oft führen persönliche Empfehlungen zur Kontaktaufnahme, nur wenige KMU suchen gezielt nach Start-up-Partnern.[3] Laut einer Unternehmensbefragung des Verbands Deutscher Maschinen- und Anlagenbau (VDMA und EBS University, 2019) setzen KMU bei der Partnersuche auf bestehende Netzwerke: 94 % der Unternehmen beziehen Empfehlungen von Kunden, Zulieferern, Partnerunternehmen, Angestellten, Verbänden oder Forschungseinrichtungen. Somit scheint trotz der digitalen Vernetzung räumliche Nähe weiterhin relevant. Dies könnte sich nun mit der Einschränkung persönlicher Kontakte im Zuge der Corona-Krise ändern.

Um den Status quo von Kooperationen zwischen Start-ups und KMU in Deutschland zu analysieren, wurde eine ökonometrische Auswertung der Ergebnisse des BDI-Familienunternehmenspanels (BDI und IfM, 2018) durchgeführt (Engels und Röhl, 2019). Insgesamt kooperierten 48 % der Befragten mit Start-ups, im Durchschnitt mit je vier Partnern. Dabei ist die Zusammenarbeit stark größenabhängig: Gut 68 % der Unternehmen mit einem Jahresumsatz von mehr als 250 Mio. Euro hatten Kooperationen mit Start-ups, aber nur knapp 43 % der KMU mit einem Umsatz bis 100 Mio. Euro. Bei den Motiven stehen die Entwicklung neuer Technologien (54 %), die Gestaltung der Digitalisierung sowie die Entwicklung von Produkten und Dienstleistungen (je 50 %) vor dem Zugang zu neuen Märkten.

Mit einer logistischen Regressionsanalyse wurde untersucht, welche Unternehmensmerkmale die Wahrscheinlichkeit beeinflussen, mit einem Start-up zu kooperieren. Es wurden zwei Modelle geschätzt. In beiden sind der di-

3 Etwa ein Drittel der in der RKW-Studie befragten Unternehmen gab an, dass ihnen aus ihrem Umfeld kein einziges Start-up bekannt sei (Wallisch, 2018).

gitale Reifegrad[4] des Unternehmens und die Exporttätigkeit Kontrollvariablen. In Modell I dient auch der Umsatz als Kontrollvariable, während in Modell II die Mitarbeiterzahl verwendet wird. Die Ergebnisse zeigen, welche Faktoren die Wahrscheinlichkeit, dass ein Unternehmen mit Start-ups kooperiert, statistisch signifikant erhöhen:

- Ist es digital reif, verdoppelt sich die Wahrscheinlichkeit.
- In Modell I verdoppelt sich die Wahrscheinlichkeit, wenn es international tätig ist.
- In Modell II hat die Internationalität keinen signifikanten Effekt, aber die Mitarbeiterzahl: Ab 500 Angestellten steigt die Wahrscheinlichkeit um das 2,3-Fache.

Aufgrund der begrenzten Zahl von Beobachtungen sind die Ergebnisse mit Vorsicht zu interpretieren. Trotz dieser Einschränkung sind folgende Schlussfolgerungen möglich:

- Digitale Unternehmen sind eher bereit, mit Start-ups zu kooperieren, als nicht-digitale. Da Start-ups häufig digital orientiert sind, bestehen in diesem Fall offenbar weniger Reibungsverluste.
- Internationale Aktivität und Unternehmensgröße haben tendenziell einen positiven Einfluss auf die Kooperationswahrscheinlichkeit. Größere Unternehmen verfügen offenbar über mehr Ressourcen, um Kooperationen zu initiieren und zu pflegen.

Die Zusammenarbeit zwischen KMU und Start-ups kann sehr unterschiedlich sein: Kunden-Lieferanten-Beziehungen, Projekt-Kooperationen, Joint Ventures, Inkubator-/Accelerator-Programme, Minderheitsbeteiligungen und Übernahmen. Die Auswertung zeigt, dass die projektbezogene Kooperation mit ca. einem Drittel am häufigsten ist. Am zweithäufigsten sind mit knapp einem Viertel Kunden-Lieferanten-Beziehungen. Joint Ventures und Minderheitsbeteiligungen spielen mit jeweils etwa einem Zehntel eine Rolle, während Übernahmen und Inkubator- oder Accelerator-Programme mit je etwa 7 % selten sind. Größere Firmen sind in allen Kooperationsformen aktiver, wobei sich Beteiligungen, Joint Ventures, Übernahmen und Inkubator-/Accelerator-Programme auf Familienunternehmen ab 1.000 Mitarbeiter:innen konzentrieren.

Die Entwicklung neuer Technologien wurde signifikant häufiger von umsatz- und beschäftigungsstarken Firmen als Motiv genannt, die ihre Situation und ihre Exportentwicklung positiv einschätzen. Auch Produkt- und Serviceentwicklung, Zugang zu neuen Märkten und Digitalisierung wurden von großen, erfolgreichen Unternehmen eher als Motiv genannt. Die Bedeutung dieser Zukunftsthemen als Kooperationsmotiv wächst. Mehr als 70 % der Unternehmen sind mit der Kooperation (sehr) zufrieden, nur 3 % unzufrieden. Allerdings sah fast die Hälfte der Firmen mit Kooperationserfahrung unterschiedliche Unternehmenskulturen als Problem. Auch aus Start-up-Sicht sind Kooperationen ein Erfolg: Laut Deutschem Startup Monitor sind drei Viertel der Start-ups mit dem Erfolg vergangener und aktueller Kooperationen mit etablierten Unternehmen zufrieden, nur 7,4 % sind nicht zufrieden (Kollmann et al., 2018).

## Fazit und Empfehlungen

Die Zusammenarbeit zwischen Mittelstand und Start-ups in Deutschland wird intensiver: Immer mehr etablierte Unternehmen sind in den letzten Jahren Kooperationen mit Start-ups eingegangen. Die Auswertung des BDI-Familienunternehmenspanels zeigt aber, dass die Kooperation mit Start-ups vor allem dann stattfindet, wenn die Unternehmen digital und an Zukunftsthemen interessiert sind. Etablierte Firmen müssen ihre Kultur für Neuerungen öffnen und Start-ups sollten mehr den persönlichen Kontakt suchen. Auf Basis der empirischen Auswertungen und Experteninterviews lassen sich Empfehlungen für eine stärkere Zusammenarbeit von etablierten Mittelständlern und Start-ups ableiten:

- Plattformangebote nutzen: Eine wachsende Zahl digitaler Kooperationsplattformen hilft bei der Vernetzung von Start-ups und KMU. Oft handelt es sich dabei um internetbasierte Start-ups, die Nutzergruppen in Kontakt bringen. Als Mittler der Vernetzung sind digitale Plattformen ein zentrales Geschäftsmodell der Digitalisierung (Demary und Rusche, 2018, 8). Trotzdem ersetzen digitale Formate nicht den physischen Austausch, sondern ergänzen und erweitern ihn.

- Auf Netzwerke zurückgreifen: Wie die Interviews gezeigt haben, spielen die bestehenden Netzwerke weiterhin eine wichtige Rolle bei der Anbahnung von Kontakten. Generell sind Intermediäre mit Kontakten sowohl im Bereich etablierter Unternehmen als auch in der Gründerszene geeignet, um KMU und Start-ups zusammenzubringen.

- Die Grenzen zwischen Industrie und Dienstleistung verschwimmen zunehmend. Industrieunternehmen, z. B. im Maschinenbau, sind heute auch Dienstleister, die eine zuverlässige Produktion durch Leistungen wie Schulung, Maschinenüberwachung, Wartung und Reparatur bieten (IW Consult, 2015). Durch die fortschreitende Digitalisierung und die Industrie 4.0 werden die Branchengrenzen in Zukunft noch durchlässiger. Folglich sollten sich produzierende Unternehmen aktiv mit der Digitalisierung und neuen serviceorientierten Geschäftsmodellen auf

4 Ein Unternehmen gilt als digital reif, wenn es die Frage „Wie sehen Sie Ihr Unternehmen im Prozess der zunehmenden Digitalisierung aufgestellt?“ mit (gut oder sehr gut) beantwortet.

Basis der von ihnen generierten Daten auseinandersetzen. Oft können Start-ups hierfür wertvolle Inputs liefern.

- Die Breitbandabdeckung und die Qualität der Mobilfunknetze wurden in den Interviews als unzureichend kritisiert, sie müssen dringend verbessert werden. Die gemeinsame Produktentwicklung erfordert z.B. sehr hohe Bandbreiten für den Datenaustausch, möglichst in Echtzeit. Dies ist in Deutschland selbst in gut versorgten Regionen nicht gewährleistet, während viele ländliche Regionen große Defizite in ihrer digitalen Infrastruktur aufweisen (Demary et al., 2019). Bis 2025 strebt die Bundesregierung eine flächendeckende Versorgung mit qualitativ hochwertigen Netzen an. Die bisher gesetzten Ausbauziele wurden jedoch verfehlt.

Weitere Empfehlungen, die sich aus den Interviews mit Expert:innen ergeben, betreffen ein innovations- und kooperationsförderndes Umfeld, die Start-up-Ausbildung im Studium, bessere öffentliche Finanzierungsinstrumente zur Förderung innovativer Gründungen (dieser Punkt wird von der Bundesregierung aktuell mit dem „Zukunftsfonds" adressiert), die Stärkung von Forschungskooperationen, Bürokratieabbau und schnellere Genehmigungsverfahren sowie integrierte Konzepte in der Wirtschaftspolitik, die auch die Standortbedingungen in den Blick nehmen.
